# Current practices in treatment of female genital fistula: a cross sectional study

**DOI:** 10.1186/1471-2393-10-73

**Published:** 2010-11-10

**Authors:** Steven D Arrowsmith, Joseph Ruminjo, Evelyn G Landry

**Affiliations:** 1 452 Union Ave. SE, Grand Rapids, MI 49503, USA; 2 Fistula Care Project, EngenderHealth, 440 9th Ave, New York, NY USA

## Abstract

**Background:**

Maternal outcomes in most countries of the developed world are good. However, in many developing/resource-poor countries, maternal outcomes are bleaker: Every year, more than 500,000 women die in childbirth, mostly in resource-poor countries. Those who survive often suffer from severe and long-term morbidities. One of the most devastating injuries is obstetric fistula, occurring most often in south Asia and sub-Saharan Africa. Fistula treatment and care are available in many countries across Africa and Asia, but there is a lack of reliable data around clinical factors associated with the success of fistula repair surgery. Most published research has been retrospective. While these studies have provided useful information about the care and treatment of fistula, they are limited by the design. This study was designed to identify practices in care that could lead to the design of prospective and randomized controlled trials.

**Methods:**

Self-administered questionnaires were completed by 40 surgeons known to provide fistula treatment services in Africa and Asia at private and government hospitals. The questionnaire was divided into three parts to address the following issues: prophylactic use of antibiotics before, during, and after fistula surgery; urethral catheter management; and management practices for patients with urinary incontinence following fistula repair.

**Results:**

The results provide a glimpse into current practices in fistula treatment and care across a wide swath of geographic, economic, and organizational considerations. There is consensus in treatment in some areas (routine use of prophylactic antibiotics, limited bed rest until the catheter is removed, nonsurgical treatment for postsurgical incontinence), while there are wide variations in practice in other areas (duration of catheter use, surgical treatments for postsurgical incontinence). These findings are based on a small sample and do not allow for recommending changes in clinical care, but they point to issues for possible clinical trial research that would contribute to more efficient and effective fistula care.

**Conclusions:**

The findings from the survey allowed us to consider clinical practices most influential in the cost, efficacy, and safety of fistula treatment. These considerations led us to formulate recommendations for eight randomized controlled trials on the following subjects: 1) Efficacy/safety of short-term catheterization; 2) efficacy of surgical and nonsurgical therapies for urinary incontinence; 3) technical measures during fistula repair to reduce the incidence of post-surgery incontinence; 4) identification of predictive factors for "incurable fistula"; 5) usefulness of urodynamic studies in the management of urinary incontinence; 6) incidence and significance of multi-drug resistant bacteria in the fistula population; 7) primary management of small, new fistulas by catheter drainage; and 8) antibiotic prophylaxis in fistula repair.

## Background

Genital fistula is an abnormal passage or opening between the genital tract and the urinary or intestinal tract. It is one of the most devastating maternal morbidities and is seen most often in Africa and Asia. There are three primary types of gential fistula:

• Obstetric, caused from prolonged or obstructed labor. This is the most common cause of genital fistula. During labor, the constant pressure of the baby's head against the vaginal and bladder or intestinal wall tissue leads to necrosis, which causes a fistula to develop. The fistula results in the uncontrolled passage of urine and/or feces into the vagina.

• Iatrogenic which results from tearing of the vaginal tissues, as in damage during obstetric or gynecologic surgery such as cesarean delivery or hysterectomy. This cause occurs less frequently than prolonged or obstructed labor.

• Violent sexual assault, including rape and forced insertion of objects into a woman's vagina. A fistula resulting from sexual violence is one example of a "traumatic gynaecologic fistula".

Women suffering from fistula live with chronic urinary and/or fecal incontinence, which often leads to social isolation, divorce, abandonment, and even abuse. Many women report feeling shame about their condition and become estranged from friends and family.

Fistulas occur in places where use and access to obstetric care is limited. While there are no sound data on the number of women living with fistula, the most commonly cited estimate is more than 2 million women living with fistula, with approximately 50,000 to 100,000 cases occurring annually, mostly in Africa, Asia, and the Arab world [1]. Further, the unmet need for fistula repair is estimated to be as high as 99% [2].

Throughout sub-Saharan Africa and parts of Asia, surgeons are working to treat fistula and its sequelae. Nevertheless, reliable data about the clinical factors associated with the success of fistula repair surgery are sorely lacking. Several attempts have been made to develop classification systems for fistula, most of which include a description of the site and size of the fistula. There is no currently agreed upon international classification system for fistula, and practitioners have issued a call for an evidence-based approach to developing a system. [3]. A few studies have been useful in identifying factors that could lead to unsuccessful fistula closure and continued incontinence, such as fistula location, fistula size, and scarring and circumferential fistula [4,5].

One study found that women are more likely to develop urinary incontinence after obstetric fistula repair if any of the following are present [6]

• Involvement of the urethra

• Small functional bladder capacity

• Large diameter of fistula

• Need for vaginal reconstruction

In an uncontrolled observational study, 55% of women reported persistent urinary incontinence after surgery, while 38% reported altered fecal continence [7].

Another retrospective study reviewed the outcome of fistula repair surgery following three different durations of catheterization: 10 days, 12 days, and 14 days. (These patients' fistulas were classified by the Goh system [8].) The authors found that patients with more damage (e.g., more vaginal scarring, a larger fistula, and greater urethral damage) were assigned to a longer catheterization period by the operating surgeon. The researchers concluded that shorter periods of catheterization may be adequate for patients with less complicated fistula, but they note that randomized clinical trials are needed to assess the effectiveness of shorter durations of catheterization [5].

One study showed the results of a surgeon who employed catheterization for possible spontaneous healing and surgical repair in less than three months from the formation of the fistula [9]. The author found that 15% of patients healed spontaneously with the catheter, with 96% of those women remaining continent. The rest of the women underwent fistula repair as soon as the fistula edge was clean (even with inflammation), with an 87% success rate (success defined as continence).

While these studies provide useful information, most of the data were gathered from retrospective record reviews and are therefore limited by the nature of the design: Only information that is available in the record can be analyzed.

At a 2005 international meeting [10], clinicians and public health professionals identified three treatment and care areas that needed more attention:

• The use of prophylactic antibiotics

• Catheter management after fistula repair

• The need for women who have had a fistula to have a cesarean delivery for subsequent pregnancies

The group also identified six research needs

1. Assessing the burden of disease attributable to fistula

2. Identifying community-level prevention measures such as delaying early childbearing and raising awareness about the need for skilled attendance at birth

3. Developing a standard classification system

4. Identifying evidenced-based practices for successful management of fistula

5. Assessing low-cost treatment methods for resource-poor settings

6. Identifying barriers to emergency obstetric care at the community, facility, provider, and patient levels

While these research topics are important, the field also needs prospective and randomized controlled trial studies that can help identify best practices for fistula surgery.

Fistula Care, a five-year project (2007-2012) funded by the U. S. Agency for International Development (USAID) and its partners are interested in identifying treatment regimens that could contribute to influencing policies and practices and that could lead to improved care for women living with fistula. In 2008, through a consultative process, fistula clinicians from 10 countries identified three priority areas that could be appropriate for clinical trials [11]:

• Use of prophylactic antibiotics

• The role of catheterization in fistula care management

• Management of urinary incontinence following fistula surgery

Before embarking on any clinical trials, Fistula Care decided to conduct a descriptive clinical review of current practices around these three issues. The goal was to identify current practices in order to frame research questions for one or more clinical trial.

## Methods

### Study Population

The study used a purposive sample of surgeons who perform fistula surgery in sub-Saharan Africa and South Asia. The authors compiled a list of 82 known surgeons working on fistula in 21 countries in sub-Saharan Africa and one country in South Asia; 12 of these surgeons (15%) reside outside of Africa and Asia and periodically serve as visiting surgeons in countries in these regions. Potential respondents included:

• Surgeons working at government and private hospitals, several of which are supported by international nongovernmental organisations (e.g., EngenderHealth/Fistula Care project, United Nations Population Fund [UNFPA])

• Contacts from two professional societies--the International Society of Obstetric Fistula Surgeons (ISOFS) and the Pan African Urologic Surgeons Association (PAUSA)

The study used no other selection criteria, such as size of hospitals where respondents work, funding, locations, or level of activity. The study proposal was reviewed and approved using EngenderHealth's standard operating procedures for ethical and technical quality in research. IRB approval was not required for the study as it did not involve any clinical procedures or use of patient records.

### Study Design

The authors were able to locate e-mail addresses for a total of 66 fistula surgeons (80%) from among the potential respondents. Letters of invitation were sent by e-mail to 62 of these surgeons. Four invitation letters were sent by messenger from Abuja, Nigeria, to fistula surgeons in Northern Nigeria who lacked e-mail access. The letter explained the rationale and design of the study-- to explore knowledge gaps regarding care for women living with fistula by gathering information about current practices in order to formulate future research questions--and included an invitation to participate. Surgeons who expressed interest received the questionnaire by e-mail and were asked to complete and return it. The questionnaire was divided into three parts, to cover each clinical area of interest. Participants were assured that all information they provided would be considered confidential. Respondents received an informed consent form for participation in the study. Participants received all study documents (letter of invitation, instructions, consent, and questionnaires) in either English or French.

### Data Analysis

The data from this study are purely descriptive (frequencies and ranges) and not amenable to formal statistical analysis. Rather, they are used as a general guide to current practices in the areas of clinical care in question, as described above. The data were reviewed for broad trends that frame current practices.

## Results

Of the 66 surgeons invited to participate in the study, 51 replied to our invitation (77%); 49 agreed to participate and were sent a questionnaire. Two invitees responded but declined to participate.

Among the 49 who agreed to participate, a total of 40 returned a completed questionnaire, yielding a final response rate of 61% (40/66). The high response rate was partly due to the authors' sending reminder e-mails to the surgeons who had agreed to participate. We do not know why we did not hear from 26 of the surgeons we contacted. The authors believe that surgeons responded to our survey for two reasons: 1) they are interested in contributing to the learning required for more evidence-based research to improve fistula services and 2) they would be interested in participating in future research. Seven of the 40 respondents are currently participating in a prospective study on fistula outcomes sponsored by EngenderHealth.

Respondents included surgeons who provide fistula care services in Asia, East Africa, Central/Southern Africa, and West Africa (Table 1). Respondents provide fistula care in a range of health care facilities, from free-standing fistula facilities to general hospitals, some under governmental and others under nongovernment organizational management.

**Table 1:** Respondents

	N	%
No. of possible surgeons to contact	82	
No. of letters of invitation sent	66	80%
No. of surgeons who agreed to participate in the study	49	74%
No. who returned completed questionnaires	40	82%
		
**Primary region where respondents provide fistula care**		
Asia	5	13%
West Africa	21	53%
Central/Southern Africa	4	10%
East Africa	10	25%
		
**Nationality**		
Country-national surgeon	32	80%
International surgeon	8	20%
		
**No. of respondents working at Fistula Care-supported sites**	18	45%
		
**Respondents' primary language**		
French	18	45%
English	22	55%

The majority of respondents (32) were country nationals, while one-fifth were international surgeons (Table 1). (Three of the eight international respondents resided in Asia or Africa; the remainder travelled periodically to provide services.) The majority of respondents (36) were male. Just over half of the respondents perform surgery at sites supported by the Fistula Care project.

The surgeons reported that they perform from 20 to 700 fistula repair cases per year, with a mean of 168 and median of 150 cases.

### Antibiotic Usage

#### Resources for Use of Antibiotics

The study found that antibiotics are usually available to fistula surgeons. Just over two-thirds said they use their hospital pharmacy formulary. Only a few reported that their patients must acquire antibiotics through private pharmacies. Several respondents commented that their supply of antibiotics comes via the organization sponsoring the fistula surgery. Over three-quarters of respondents reported they have access to quinolones and to aminoglycosides, and more than half have third-generation cephalosporins (see Table 2). Only two respondents reported access to extended spectrum ("rescue") antibiotics like imipenem for use in a setting of bacterial multi-drug resistance. Factors affecting the availability of antibiotics include market-driven factors (drug availability and hospital budget), hospital formulary, or government essential drug lists.

**Table 2:** Antibiotic Practices for Fistula Surgery

	N	%
Antibiotics usually available for pelvic surgery *		
Aminoglycosides (e.g., gentamicin/tobramycin)	31	78%
Quinolones (e.g., ciprofloxacin)	31	78%
Second-generation cephalosporins (e.g., cefuroxime)	15	38%
Third-generation cephalosporins (e.g., ceftriaxone)	22	55%
"Rescue" antibiotics for multi-resistant organisms (e.g., imipenem)	2	5%
		
**Factors influencing availability of antibiotics^**		
Market factors: drug availability and hospital budget	12	30%
Hospital formulary set by administration	11	28%
Ministry of Health's essential drug list	8	20%
Donation from partners	4	10%
Other	4	10%
No response	1	3%
		
**Use of prophylactic antibiotics^**		
For every fistula case	23	58%
For selected fistula cases	14	35%
Never	2	5%
No response	1	3%
		
**Most important factor in choice of prophylactic antibiotics^**		
Recommendations of medical literature	9	23%
Surgeon training in vesicovaginal fistula surgery	9	23%
Availability	7	18%
Personal choice	5	13%
Cost	2	5%
Does not use prophylactic antibiotics	1	3%
Other	7	18%

* Multiple responses allowed; percentages exceed 100%.

^ Exceeds 100% due to rounding.

#### Current Practices in Systemic Antibiotic Prophylaxis

Clinicians debate the definition of antibiotic prophylaxis; for the purposes of this study the term means a single dose of antibiotics given before surgery to prevent the development of infection.

When asked about antibiotic use as prophylaxis, more than half reported using antibiotics on every surgical case, and only two respondents reported that they do not use antibiotics routinely in fistula care (Table 2). However, some internal inconsistency was noted in the responses. The questionnaire included the response choice "I don't use prophylactic antibiotics" for five different questions, and the number of respondents who reported non-use of antibiotics ranged from zero to five.

**Figure 1:**
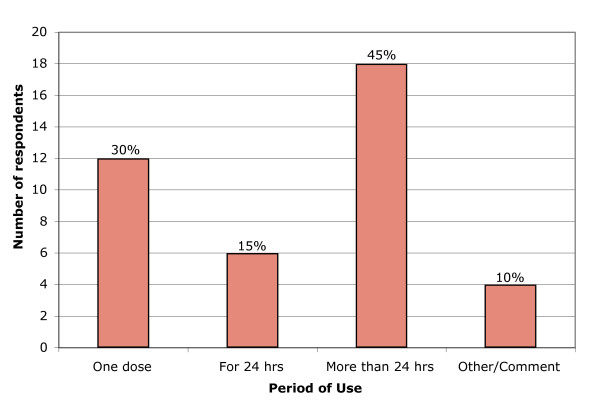
**Length of Prophylactic Antibiotic Use**. Description: length of prophylactic use reported by respondents. Y axis: Number of respondents. X axis, Period of use (one dose, for 24 hours, More than 24 hours, other comments).

Overall, the majority of respondents reported that their patients receive prophylactic antibiotics. However, there was little agreement as to what type of antibiotic and regimen to use. The most commonly chosen single agent was an aminoglycoside (e.g., gentamicin). Nearly three-quarters of the respondents reported that they start patients on antibiotics before surgery. The duration of antibiotic use for "prophylaxis" varied widely (see Figure 1): 12 respondents report they give a single preoperative dose, and 18 respondents continue antibiotics more than 24 hours after surgery. (By clinical definition, continuation of antibiotics beyond 24 hours is considered empiric antibiotic therapy, not prophylaxis.) About half of the surgeons (18) reported having a routine antibiotic choice for all fistula patients; most of the remaining respondents (16) stated that the choice of antibiotic was made on an individual basis with each case.

#### Rationale for Use of Prophylactic Antibiotics

Many respondents said that their routine use of antibiotics is based on either the medical literature or their clinical training in fistula surgery (Table 2). But "personal experience" and "availability" were also common reasons given, often by both those who do not use prophylactic antibiotics and those who do. Cost to the patient was not a significant factor in decision making.

### Urethral Catheter Management

#### Resources for Use of Urethral Catheter

Almost two-thirds of respondents reported that their hospitals purchase catheters locally. One-third receive catheters from donors; only four respondents reported requiring patients to purchase catheters before surgery. Approximately three-quarters of respondents said that their choice of catheter is not affected by availability.

#### Current Practices in Catheter Management

Catheter management practices varied widely among respondents (Table 3). Some surgeons stated that they prefer big catheters (20F as a routine choice), while others prefer small 12F catheters. The average preferred size was a 16-18F tube, with nearly all preferring the standard Foley catheter. Almost half preferred latex catheters; one-third said that the composition of the catheter does not matter.

**Table 3:** Role of Catheterization

**Ideal choice of catheter size (French) for routine urinary fistula repair**		
Minimum	12	
Maximum	20	
Average	17	
No response	6	
	**N**	**%**
**Ideal choice of catheter type^**		
Standard Foley catheter	37	93%
Straight catheter without balloon	1	3%
Specialty catheter	1	3%
No response	1	3%
		
**Routine catheter drainage**		
Closed drainage (catheter attached to closed drainage bag)	26	65%
Open drainage (catheter drains into basin, bottle, or bucket)	12	30%
No response	2	5%
		
**Patients remain on bed rest until the catheter is removed^**		
Always	7	18%
Sometimes	6	15%
Never	23	58%
No response	4	10%
		
**Bladder training/clamping the catheter carried out before the catheter is removed^**		
Always	5	13%
Sometimes	11	28%
Never	23	58%
No response	1	3%
		
**After the catheter is removed, a program of bladder training begins**		
Always	6	15%
Sometimes	22	55%
Never	10	25%
No response	2	5%
		
**Patients who are unable to urinate are taught intermittent self-catheterization ^**		
Yes	15	38%
Sometimes	9	23%
No	15	38%
No response	1	3%

^ Exceeds 100% due to rounding.

Nearly one-third of respondents reported open drainage of the catheter into a basin (Table 3); nearly all respondents reported that they use a catheter with a balloon. Only one-fifth of the surgeons reported they require bed rest until the catheter is removed; two-thirds never make patients stay in bed until the catheter is removed (Table 3).

**Figure 2:**
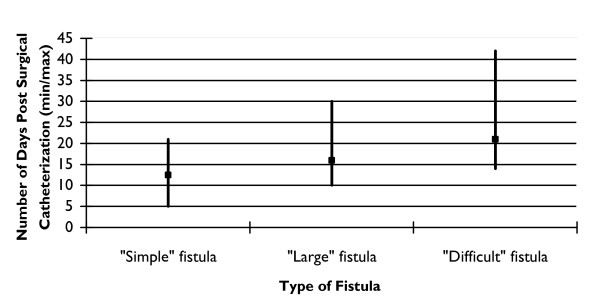
**Duration of Catheter Use by Type of Fistula**. Description: duration of catheter use by type of fistula reported by respondents. Y axis: Number of Days Post Surgical Catheterization (minimum/maximum). X Axis: Type of fistula: Simple fistula, Large fistula, Difficult fistula.

Duration of catheter use showed the greatest range of practice. Respondents were asked "How long are catheters left in place after fistula repair?" for three types of fistula--simple, large, and difficult. (Because there is no standard terminology for the description of fistula the terms "simple," "large," and, "difficult" were left to the respondents to interpret.) For a "simple" fistula, the average was 12 days, ranging from a minimum of five days to 21 days (see Figure 2). For a "difficult" fistula, respondents reported leaving the catheter indwelling for as many as 42 days as a routine (the average was 21). For a "large" fistula the time was intermediate, with a range of 10-30 days and an average of 17. About one-quarter of surgeons routinely performed a dye test before removing the catheter.

Respondents were asked about their practice in bladder "training": intermittent clamping of the catheter to allow the bladder to fill and distend to some degree before catheter removal. (The precise definition of this term was not clear in the questionnaire and was left to the interpretation of the respondent.) Less than half of the respondents reported that they occasionally begin clamping the catheter before removal to begin bladder training (Table 3). After the catheter is removed, about three-quarters perform bladder training of some sort for at least a part of their patient population (Table 3).

For patients who are unable to urinate after the catheter is removed, more than half of surgeons said that they instruct the patients in intermittent self-catheterization (Table 3). When a patient requires prolonged catheterization, about one-fifth of respondents said that they discharge a patient to go home with the catheter still indwelling.

#### Catheter Management by Nursing Staff

Most respondents reported that nursing staff in their facilities flush catheters; just over one-third reported that nurses replace the catheter if it becomes blocked, without the need for specific direction from the physician.

#### Catheterization as Primary Treatment of Select Fistula

A small number of women present with small, "fresh", and/or simple fistula that can be treated with catheter drainage alone. (Within the fistula community, the term "fresh" is understood to refer to a fistula occurring within the last 3-6 months.) One provider reported managing nearly one-third of cases in this way, while others reported never seeing patients who would fit the criteria to employ this approach. Many respondents agreed that primary treatment of fistula with a catheter can be a successful therapy in select women, but that the ideal patient for this treatment is simply not seen often enough.

### Management of Urinary Incontinence after Fistula Repair

#### Resources for Diagnosis of Urinary Incontinence

The majority of respondents said that they diagnose post-surgery urinary incontinence on simple clinical grounds: About three-quarters use a combination of history, a physical examination, and a negative dye test, while one-fifth use a physical examination and history. Only a few of the respondents reported ever using urodynamic studies (UDSs) (see Table 4). Study respondents were asked to self-report a rough estimate of the incidence of incontinence in their patients after successful closure of an obstetric fistula. These estimates ranged from as low as 0.5% to as high as 30%, and overall, these self-estimates averaged 11%.

**Table 4:** Management of Urinary Incontinence after Fistula Repair

	N	%
**Method for diagnosing incontinence after fistula surgery^**		
By history and physical examination, including dye test	29	73%
By history and physical examination, without dye test	8	20%
By urodynamic studies	1	3%
No response	2	5%
		
Operative procedures performed to reduce the risk of postoperative SUI*		
Urethral lengthening	17	43%
Bulbocavernosus sling (Browning)	14	35%
Bladder neck suspension	11	28%
Medial thigh fascio-cutaneous flaps	3	8%
Other	11	28%
No response	1	3%
		
Non-surgical treatment provided for incontinence after fistula repair*		
Pelvic floor exercises	30	75%
Anticholinergic medications (buscopan, oxybutinin, etc.)	21	53%
Bladder training	17	43%
Urethral plugs	5	13%
Peri-urethral injection (autologous fat, collagen, microspheres)	2	5%
Other	5	13%
No response	3	8%
		
Surgical treatments provided for urinary incontinence after surgery*		
Pubovaginal sling surgery	19	48%
Bladder neck suspension	18	45%
Vaginal tape procedure	6	15%
Other	10	25%
No response	2	5%
		
Treatments/care provided to patients who have failed surgical treatment for stress incontinence*		
Counseling	28	70%
Long-term pelvic floor exercises	22	55%
Urinary diversion	14	35%
Urethral plugs	7	18%
Other/comment	3	8%

* Multiple responses allowed; percentages exceed 100%.

^ Exceeds 100% due to rounding.

#### Importance of Post-Surgery Incontinence

Three-quarters of surgeons believe that post-surgery incontinence is important because the condition is common and it seriously affects the woman's quality of life. Consequently, respondents routinely include it in the counseling provided to patients before repair surgery.

#### Intra-Operative Predictive Factors for Post-Surgery Incontinence

Respondents ranked a list of physical examination findings in order of relevance (1 through 8) as predictors of urinary incontinence after fistula repair. Three factors--urethral length, degree of urethral damage, and presence of pelvic prolapse--were the surgeons' top three choices. Pelvic prolapse was considered the least important of these three. About one-fifth of the respondents said they would consider not performing fistula repair if they believed the patient was likely to have severe incontinence after surgery.

#### Intra-Operative Techniques for Preventing Post-Surgery Incontinence

According to most respondents, certain technical interventions done during the fistula repair itself can reduce the risk of post-surgery incontinence. Most popular among these procedures was lengthening of the urethra, followed by techniques classically used in wealthy nations to treat stress incontinence such as slings and bladder neck suspensions (Table 4).

#### Current Practices in Nonsurgical and Surgical Treatment

Respondents reported use of nonsurgical treatments for post-repair incontinence (Table 4). Just over three-quarters of respondents recommend pelvic floor exercises, just over half prescribe medications, and less than half institute bladder training. Few had tried injection of periurethral bulking agents or use of urethral plugs.

#### Surgical Treatments Following Repair

Two-thirds of surgeons said that they had received formal training in surgical therapy for urinary incontinence. The most popular individual surgical procedure was the pubovaginal sling and bladder neck suspension, performed by about half of the respondents (Table 4). A few respondents reported using tension-free vaginal tape, which involves insertion of a loose sling of plastic mesh.

#### Definition and Management of Treatment "Failure"

The questionnaire asked respondents how they define success of fistula treatment surgery. One-quarter define surgical success anatomically, while the majority favor a functional definition of success, meaning that the patient needs to be dry after surgery for the intervention to be considered successful.

On average every year, each surgeon sees about 13 women (range 0-50) with post-surgery incontinence for whom the initial intervention, either surgical or nonsurgical, failed. Second-line therapy is distributed over a range of options. Nonoperative treatments were common: The majority offer counseling, about half offer training in long-term pelvic floor exercises, and a few provide urethral plugs (Table 4). Less than half said that they offer urinary diversion as a treatment option for women failing all other intervention. About half said that they were not trained in diversion procedures.

## Discussion

### Study Limitations

The study was an attempt to describe in broad strokes current practices in three defined dimensions of fistula care. The sample size and sampling method did not allow for analysis beyond descriptive statistics. Because the community of fistula surgeons is small, the researchers could not randomly select a subgroup of respondents. Because the questionnaire used multiple choice and pre-coded responses, the study was not able to capture all aspects of clinical practice. While most questions included an "other category" where respondents could provide other responses, this feature was not used by all respondents. A further limitation of the design is that self-reporting may reflect ideal rather than actual practice. While the findings do not allow the authors to recommend changes in clinical care, the research is nonetheless important because it is the first published study on current clinical practice in fistula care. This research provides guidance about future strategies for research and fistula care.

### Current Practices

#### Antibiotic Use

Antibiotic therapy is expensive and 95% of the study respondents use antibiotic prophylaxis; thus, this area is fertile ground for study (Table 1). It is difficult to discern from the data any patterns in the choice of antibiotics or duration of therapy. Two-thirds of respondents reported use of empiric, rather than prophylactic, antibiotic therapy. Certainly, as training curricula for fistula surgeons are being developed, guidance for antibiotic use needs to be standardized. One of the few clinical trials in the literature regarding peri-operative care in the fistula patient specifically addressed antibiotic use in the fistula population [12]. This study, from Benin, found no benefit from prophylactic antibiotics. If this is true, antibiotic use may be wasting significant resources.

Adaptation of guidelines from Western nations is a potential approach. Major professional organizations with expertise in pelvic surgery, such as the American College of Obstetricians and Gynecologists [13] and the American Urological Association [14] have well-defined policies regarding antibiotic use. Furthermore, research has examined the use of antibiotics with "contaminated" wounds and other types of pelvic surgery; thus, there is probably little to be gained from studies specifically focused on fistula repair. Are separate clinical trials for fistula repair necessary if large studies have already been performed for other types of pelvic surgery? The authors believe that a large, complex, and expensive trial is not needed.

In this study, a few surgeons reported having access to expensive extended spectrum antibiotics to treat infection caused by multi-drug resistant organisms. In recent fistula care outreach camps in West Africa, the prevalence of multi-drug resistance has been alarmingly high [15,16 ]. If fistula care centers do not have the antibiotics to treat these infections, patients are at high risk for death from sepsis. The fistula care community may need to re-evaluate the provision of antibiotic support. A clinical trial could perform urine culture at admission in a broad range of centers; such research would help determine the prevalence and risk of multi-drug resistance.

#### Urinary Incontinence after Fistula Repair

This is clearly another area where more research is needed. The survey found that surgeons do not commonly use UDSs to manage incontinence after fistula repair. Research could determine if UDSs are helpful in diagnosing fistula, identifying appropriate therapies, and improving outcomes. However, urodynamics hardware and consumables are expensive, and training staff to perform these complex tests would be time-consuming and costly.

According to one study, presented at the International Society of Obstetric Fistula Surgeons 2^nd ^Annual Meeting in Nairobi (2009), approximately one-third of women have significantly impaired bladder emptying after fistula repair (Williams G: Diagnosis and management of incontinence after fistula surgery at Addis Ababa Fistula Hospital, unpublished). In this study surgeons reported using a wide range of treatments for urinary incontinence after fistula repair. Both the nonsurgical and surgical therapies identified in this study were developed for a completely different condition--namely, stress urinary incontinence (SUI) from loss of pelvic floor support after childbirth. Clinicians do not know if any of these treatments, invasive or otherwise, are of benefit for incontinence following fistula repair. A prospective clinical trial would enrol women with urinary incontinence after fistula repair and then tabulate the results for the various therapies used. In such a trial, a large number of patients would be necessary to case-match for their injuries before they are randomized to different treatment modalities. If clear differences in outcomes emerge, then the more efficacious interventions can be recommended to the community of fistula surgeons. Such a study might lead to other trials to refine the field's understanding of urinary incontinence after repair surgery. Researchers could also assess the effectiveness of treatments in combination to see if outcomes improve.

Many respondents agreed that the way in which repairs is done affects the risk of incontinence after surgery. But clinicians do not know which types of repair are most effective. Only a clinical trial will answer this question.

The study included questions about urinary diversion in patients with untreatable incontinence after fistula repair. Delineating the exact profile of such an incurable condition is an attractive possibility for a clinical trial. Such cases are relatively few, however. Thus it would be time-consuming and costly to recruit enough patients to establish statistical significance.

#### Catheter Management

Duration of catheter use after repair is the single most important issue in terms of possible impact. For most facilities, length of stay is determined by duration of catheter drainage. Length of stay may be the primary determinant of surgical cost and of capacity for care. Shorter stays mean more rapid turnover and, in turn, more women receiving the opportunity for care. If everyone were leaving catheters in place for 21 days, it would be difficult to argue that perhaps this length of time could be reduced. However, the survey showed routine catheter drainage strategies ranging from five to 42 days. If the five-day patients do as well as the 42-day patients, the implications are enormous. If duration of catheterization were shortened, capacity would instantly increase without any capital investment. A clinical trial to address this issue is much needed.

## Conclusions

The primary objective of this study was to identify issues in fistula care which would benefit from clinical trials. In order to be the subject of a clinical trial, a practice needs to be pervasive and have significant implications in terms of costs, time, efficiency, or outcome. This survey provides a snapshot of current fistula practice across a wide swath of geographic, economic, and organizational conditions. The results point out surprising degrees of consensus in some areas, while highlighting the tremendous disparity of practices in other areas.

Based on the findings from this study and on discussions at two international meetings on fistula care [10,11], the authors have identified eight possible topics for clinical study: These topics, in order of priority, are:

1. Efficacy/safety of short-term catheterization after fistula repair

2. Efficacy of surgical and nonsurgical therapies for urinary incontinence after fistula repair

3. Technical measures during fistula repair to reduce the incidence of post-surgery incontinence

4. Identification of predictive factors for the "incurable fistula"

5. Usefulness of UDSs in management of urinary incontinence after fistula repair

6. Incidence and significance of multi-drug resistant bacteria in the fistula population

7. Primary management of small, new fistulas by catheter drainage

8. Antibiotic prophylaxis in fistula repair (choice of agent, onset and duration of therapy)

In addition, the authors encourage the fistula care community to do the following:

1. Emphasize training in the use of antibiotics (what is prophylactic antibiotic use, what is empiric therapy), the problem of multi-drug resistant organisms, and the need for "rescue" antibiotic therapy.

2. Produce a technical guidance document based on a literature review that could be used to support this training. Draw upon policies set by major medical organizations when preparing this document. Include information that antibiotic therapy may not be beneficial.

### Next Steps

EngenderHealth, the implementing agency for Fistula Care, is seeking funding to conduct a clinical trial on short-term catheterization, the most important priority identified in this paper. The organization will form an advisory group composed of clinical experts in fistula treatment, including principal investigators from potential study sites, to jointly design a clinical protocol for the study.

## Competing interests

The authors declare that they have no competing interests.

## Authors' contributions

SDA and JR made principal contributions to the study design. SDA analyzed the data and wrote the paper. JR was involved in drafting the manuscript and revising it critically, as well as participating in the analysis. EL participated in the design and coordination and helped draft the manuscript. All authors read and approved the final manuscript.

## Pre-publication history

The pre-publication history for this paper can be accessed here:

http://www.biomedcentral.com/1471-2393/10/73/prepub
